# Stimulation of the Social Brain Improves Perspective Selection in Older Adults: A HD-tDCS Study

**DOI:** 10.3758/s13415-021-00929-2

**Published:** 2021-07-21

**Authors:** A. K. Martin, G. Perceval, M. Roheger, I. Davies, M. Meinzer

**Affiliations:** 1 UQ Centre for Clinical Research, The University of Queensland, St Lucia, Australia; 2 Department of Psychology, The University of Kent, Canterbury, UK; 3 Department of Psychology, Soochow University, Suzhou, China; 4 Department of Neurology, University of Greifswald, Greifswald, Germany; 5 Queensland Brain Institute, University of Queensland, St Lucia, Australia

**Keywords:** Perspective taking, Aging, Right temporoparietal junction, Dorsomedial prefrontal cortex, Self-reference effect, Social cognition

## Abstract

There is evidence for dissociable, causal roles for two key social brain regions in young adults. Specifically, the right temporoparietal junction (rTPJ) is associated with embodied perspective taking, whereas the dorsomedial prefrontal cortex (dmPFC) is associated with the integration of social information. However, it is unknown whether these causal brain-behaviour associations are evident in older adults. Fifty-two healthy older adults were stratified to receive either rTPJ or dmPFC anodal high-definition transcranial direct current stimulation in a sham-controlled, double-blinded, repeated-measures design. Self-other processing was assessed across implicit and explicit level one (line-of-sight) and level two (embodied rotation) visual perspective taking (VPT) tasks, and self-other encoding effects on episodic memory. Both rTPJ and dmPFC stimulation reduced the influence of the alternate perspective during level one VPT, indexed by a reduced congruency effect (difference between congruent and incongruent perspectives). There were no stimulation effects on level two perspective taking nor self-other encoding effects on episodic memory. Stimulation to the rTPJ and dmPFC improved perspective selection during level one perspective taking. However, dissociable effects on self-other processing, previously observed in young adults, were not identified in older adults. The results provide causal evidence for age-related changes in social brain function that requires further scrutinization.

**Supplementary Information:**

The online version contains supplementary material available at 10.3758/s13415-021-00929-2.

## Introduction

Social cognition declines in advanced age (Moran et al., 2012) and is associated with impaired social functioning (Bailey et al., 2008). Age-related differences are apparent for higher-order cognitive and affective theory of mind tasks (Henry et al., 2013), but also lower-order social cognitive processes, such as the ability to integrate and distinguish between representations relevant to both the self and others (Martin et al., 2019a).

Self-other processing can be measured across several cognitive domains. Visual perspective taking (VPT) refers to the ability to understand a visual scene from the egocentric and allocentric perspective and how these may differ. Both implicit and explicit processes are thought to be involved, in line with the two-system theory of human social cognition (Apperly & Butterfill, 2009). Implicit VPT refers to the automatic calculation of another’s perspective without any prompting to consider the alternate perspective. Visual scenes are presented with an agent or non-agentic control (arrow, light, etc.), and participants must respond from their own perspective as to how many target stimuli are visible. Crucially, the number of target stimuli are congruent or incongruent with the hypothetical number of stimuli that would be visible from the other agent’s perspective. An implicit effect is identified when response times are slower during the incongruent trials compared with the congruent trials, but only when an agent is in the scene and not the nonagentic control (Apperly & Butterfill, 2009; Martin, Perceval, et al., 2019a). Explicit perspective taking requires switching between egocentric and allocentric perspectives. Two forms have been identified and are often labelled level one and level two VPT. Level one requires judgements based on *“What is visible from another perspective?”* and is thought to rely on a line-of-sight strategy. Level two VPT requires judgements on *“How something is seen from another perspective?”* and crucially is thought to rely on an embodied rotation of the egocentric perspective into the alternate perspective (Kessler & Rutherford, 2010; Michelon & Zacks, 2006). In both forms of VPT, when the alternate perspective is incongruent this often leads to interference. The extent of that interference is a reflection of the participant's ability to attend to the relevant perspective and inhibit the alternative. This is often referred to as *perspective selection.*  

Self-other processing also is relevant in the domain of episodic memory. For example, words encoded in relation to the self are more accurately recognized than those encoded in relation to someone else—a phenomenon known as the self-reference effect (SRE). In contrast to the VPT tasks, there is no requirement for online control of self-other representations and therefore no congruency effects reflecting the requirement to select between competing online task demands. Rather, a SRE may reflect greater attention to self-relevant stimuli, possibly due to a self-attention network (Cunningham, 2016; Humphreys & Sui, 2016), during the encoding of episodic memories.

Self-other processing changes across the healthy lifespan. For example, it has previously been shown that older adults are slower at adopting an allocentric perspective during both line of sight (level one) and embodied rotation (level two) perspective taking (Martin, Perceval, et al., 2019a). It also has been suggested that older adults rely less on embodied strategies and more on visual processing across a range of social cognitive measures (Costello & Bloesch, 2017), including spatial representation in relation to other agents in a scene (Committeri et al., 2020). Episodic memory also shows age-related decline (Levine et al., 2002), and specific to self-other processing, the self-reference effect in episodic memory (the bias toward remembering items encoded in relation to the self) is attenuated in older adults (Gutchess, Kensinger, Yoon, & Schacter, 2007b).

However, despite considerable research addressing social cognitive differences in young and older adults, there is a paucity of research into how the social brain changes across the lifespan. For example, functional neuroimaging changes have been associated with age-related declines in executive functioning (Fjell et al., 2017) and episodic memory (Fjell et al., 2016), but little is known about brain changes associated with social cognition. One prominent hypothesis for brain aging posits that a dedifferentiation, or reduced modularity of the brain, contributes to reduced cognitive performance (Goh, 2011; Grady, 2012). Although the brain regions consistently correlated with social cognitive processes often are labelled the social brain, substantial evidence exists for distinct roles in young individuals. One key hub of the social brain is the right temporoparietal junction (rTPJ) with several existing theories on its role in social cognition. The rTPJ is thought to be important in self-other processing with evidence for a role in self-other distinction (Bardi et al., 2017; Santiesteban et al., 2012; Schurz et al., 2013) or inhibition of self-related cognitive processes (Payne & Tsakiris, 2017; Soutschek et al., 2016). More specifically, the rTPJ may have a role in embodied processes, such as embodied egocentric rotation in order to take an alternate perspective in a visual scene (Martin et al., 2018; Martin et al., 2020; Wang et al., 2016).

Another region consistently correlated with social processes is the dorsomedial prefrontal cortex (dmPFC). The dmPFC is thought to facilitate the integration of social information, whether it be in the form of integrating across sensory domains (Ferrari et al., 2016) or self-other representations (Martin, Dzafic, et al., 2017a). However, despite general theories for dedifferentiation of brain-behaviour association in older adults, little is specifically known regarding social brain changes across the lifespan. This can be investigated by using noninvasive brain-stimulation techniques, such as transcranial direct current stimulation (tDCS), which establish causal brain-behavior relationships in social cognition. During tDCS, a weak electrical current (typically 1-2 mA) is administered to the brain, which modulates cortical excitability in underlying brain regions and activity in functionally connected brain regions (Meinzer et al., 2012). Moreover, recently introduced high-definition (HD-tDCS) montages allow the targeting of specific networks with high spatial precision (Martin et al., 2018; Martin, Huang, et al., 2017b; Villamar et al., 2013), which resulted in regionally and task-specific behavioral modulations (Gbadeyan, McMahon, et al., 2016a; Martin et al., 2018). For example, in a previous study from our group (Martin et al., 2018), HD-tDCS confirmed dissociable, causal roles of the dmPFC and rTPJ in healthy young adults. Specifically, excitatory (anodal) stimulation to the dmPFC resulted in greater influence of the allocentric perspective during egocentric perspective taking in both level one and two visual perspective taking tasks and the removal of the self-reference effect in episodic memory. Anodal stimulation to the rTPJ specifically reduced the influence of the egocentric perspective when adopting an alternate perspective during a level two visual perspective taking task. These effects have been directly replicated in young adults from a different cultural background (Martin, Su, & Meinzer, 2019b), which supports previous research in regards to causal roles of the dmPFC (Ferrari et al., 2016) and the rTPJ (Santiesteban et al., 2012; Santiesteban et al., 2015; van Elk et al., 2017; Wang et al., 2016).

Despite the recent focus on using tDCS to study the social brain (Sellaro et al., 2016), no previous study has investigated its use in healthy older adults. This is of particular interest as age-associated functional brain re-organization may result in different stimulation effects compared to younger cohorts (Perceval et al., 2016). The current study investigated whether the dissociable effects of anodal HD-tDCS to the dmPFC and the rTPJ in self-other processing, identified in young adults, would be replicated in healthy older adults. Therefore, we expect to identify a greater influence from the allocentric perspective during both level one and level two egocentric perspectives taking and removal of the self-reference effect in episodic memory after dmPFC stimulation and a reduction in interference from the egocentric perspective during level two allocentric perspective taking. However, as age-related differences in self-other processing have been well documented (Gutchess, Kensinger, Yoon, & Schacter, 2007b; Martin, Perceval, et al., 2019a), and baseline differences in behavioral performance may influence subsequent stimulation response (Martin, Meinzer, et al., 2017c; Martin, Su, & Meinzer, 2019b), stimulation of the social brain in older adults may affect self-other processing in a unique manner to that previously observed in young adults.

## Methods

### Participants

A total of 52 healthy older adults (55-79 years) were stratified by sex and assigned to either sham-controlled dmPFC or rTPJ HD-tDCS double-blind crossover studies. Sample size was adequate to detect medium-sized effects (Cohen’s f = 0.30) with power at 80% and alpha at 0.05. Stimulation order was balanced at both stimulation sites. The groups were matched for sex (13 M/F at each site) and age (dmPFC/rTPJ: 66.7 yr/65.4 yr), BF_10_ = 0.34. The groups were comparable on scales of autism, anxiety, and depression symptoms and on neuropsychological functioning (see Table 3 for details). All participants were tDCS naïve, were not currently taking psychoactive medications or substances, and had no history of neurological or severe mental health issues. All participants provided written consent, completed a safety screening questionnaire before testing, and were compensated for their time with a small monetary compensation. The study abided by the ethical standards as per The Declaration of Helsinki (1991; p1194). Ethical clearance was granted by The University of Queensland.

### Baseline Testing

All participants completed baseline cognitive assessment to ensure the two groups (dmPFC and rTPJ stimulation sites) were comparable and that all participants were within expected age-related norms. As in our previous studies (Martin et al., 2018; Martin, Dzafic, et al., 2017a; Martin, Huang, et al., 2017b; Martin, Su, & Meinzer, 2019b), tests included the Stroop test, phonemic, and semantic verbal fluency. These were completed immediately following the first stimulation session. Following the second session, participants completed the computerized CogState cognitive battery (www.cogstate.com), including the tests: International shopping test, identification test, one-back, two-back, set-switching test, continuous paired associates learning test, social-emotional cognition test, and the international shopping test-delayed recall. The CogState battery was chosen, because it is repeatable, easy-to-administer, user-friendly, with good test-retest reliability (Cole et al., 2013), validity (Mielke et al., 2015), and is sensitive to age-related cognitive decline (Lim et al., 2013).

Participants also completed the Autism Spectrum Quotient (ASQ; Baron-Cohen, Wheelwright, Skinner, et al., 2001b) and Hospital Anxiety and Depression Scales (HADS; Zigmond & Snaith, 1983).

### Transcranial Direct Current Stimulation

Stimulation was delivered using a one-channel direct current stimulator (DC-Stimulator Plus, NeuroConn). The anode was a small circular electrode (2.5 mm in diameter), and the return electrode was a concentric ring (inner/outer diameter: 9.2/11.5 cm), placed equidistantly around the central electrode. At the rTPJ, the cathode was slightly smaller (inner/outer diameter: 7.5/9 cm) than at the dmPFC due to the position of the right ear. Modelling of electrical current flow has been conducted previously for this montage (Martin et al., 2018; Martin, Huang, et al., 2017b) and demonstrated focal delivery to the target region. Safety also has been demonstrated (Gbadeyan, Steinhauser, et al., 2016b). Electrodes were held in place with electroconductive gel (Weaver Ten20 conductive paste) and an EEG cap to ensure consistent adhesion to the skin. The dmPFC was located 65% of the distance from FZ toward the FPz using the 10-20 EEG system. The rTPJ was located at CP6 of the EEG 10-20 system. In both stimulation sites, the current ramped up to 1 mA over 8 seconds and ramped down over 5 seconds. In the “sham” condition, the current was maintained at 1 mA for 40 seconds, whereas in the active condition, the current was maintained at 1 mA for 20 minutes. Researchers were blinded to the stimulation condition using the “study-mode” of the DC-Stimulator (a preassigned code programmed into the stimulator). Participants also were blind to the stimulation condition. To avoid carryover effects, testing sessions were at least 72 hours apart.

### Visual Perspective Taking Test

**Fig. 1:**
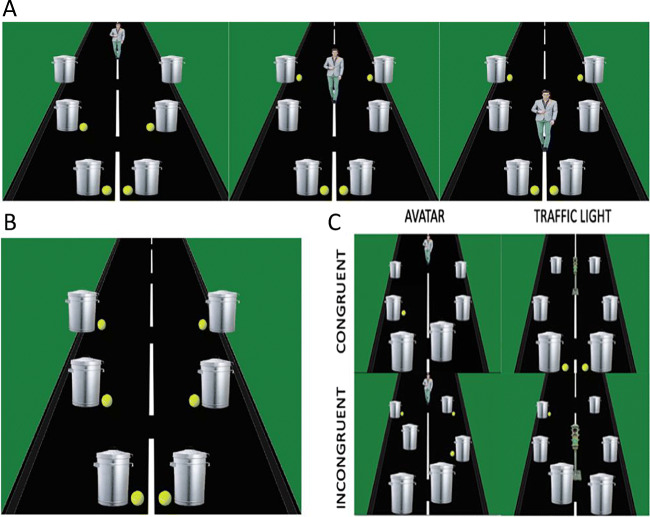
**Visual Perspective Taking (VPT) Task**. **a** Three possible locations of the avatar (or traffic light). **b** Six possible locations of the tennis balls. One to four balls were presented in any of the six locations. **c** Examples of congruent and incongruent conditions for both the avatar and the traffic light

The visual perspective task (VPT; Martin, Perceval, et al., 2019a) involved three separate tests measuring level one VPT (implicit and explicit) and level two VPT (explicit). All tests involved a street scene with tennis balls, rubbish bins, and either a human avatar or a traffic light directly in front of the gaze of the subject at one of three positions on the street: far, middle, or near. Perspective taking was performed in the extrapersonal space (Michelon & Zacks, 2006). A detailed schematic of the VPT task is presented in Fig. 1. The traffic light was used as a directional control that should direct attention in a similar manner to the human avatar, but crucially without the ability to hold a perspective of the scene, which was particularly of interest in the implicit VPT task (Apperly & Butterfill, 2009; Samson et al., 2010). Participants were instructed to answer, “How many tennis balls they/other could see?” as quickly and as accurately as possible. The stimuli remained on the screen until a response was recorded. A fixation cross was presented for 500 ms before the stimuli. For the level one and level two VPT, the word “you” or “other” was presented for 750 msec before the presentation of the scene. Participants were informed that tennis balls would be hidden from the avatar's view if a rubbish bin occluded the view or if the tennis ball was behind the avatar. If the traffic light was present, the participants were instructed to imagine the light radiating out from the traffic light toward the subject and to answer how many tennis balls the light would directly hit. Again, if a bin occluded the light or if the ball was behind the traffic light, then the light would not directly hit the ball. The test consisted of 176 trials. In 50% of the trials (n = 88), a human avatar was present, and in 50% of the trials a traffic light was present. The trials were further separated (50% each, resulting in 44 trials in each condition) by whether the number of balls seen by the subject was congruent or incongruent with that of the human avatar’s view or the number of tennis balls the light would directly hit. This resulted in four conditions: avatar congruent, avatar incongruent, light congruent, light incongruent. All conditions were balanced for number and location of tennis balls and whether the agent was near, middle, or far in the visual scene (Fig. 1). Each VPT had four counterbalanced versions, and participants were presented with different versions in each session. All tests were completed in the following order: level one implicit, level one explicit, and level two explicit.

#### Visual Perspective Task – Level One Implicit

In the first test, participants were instructed to respond as fast and accurately as possible with “How many tennis balls can you see?” The answer was always between one and four with the response buttons clearly marked on the keyboard. The task was considered an implicit test, because participants were not directed to consider the perspective from the perspective of the avatar in the scene and were only required to answer from the egocentric perspective.

#### Visual perspective task – Level one explicit

In the level one explicit task, participants were required to take either an egocentric perspective or the allocentric perspective from the avatar or light and answer how many tennis balls could be seen. There were four possible responses for each condition, with one to four tennis balls for the egocentric judgements allocentric congruent conditions. To maintain four choices for the allocentric incongruent condition, without increasing the number of balls in the scene, scenes with zero balls visible to the avatar/light were included. Therefore, answers in this condition were from zero to three.

#### Visual Perspective Task – Level Two exPlicit

In the level two explicit VPT task, participants were again required to take either an egocentric perspective or the allocentric perspective of the avatar or light. However, this task required making a judgement on ”how” the subject or other avatar views the scene, by asking them “whether they/other could see/light would shine on, more balls on the left, right, or equal number on each side of the road?” All conditions had three possible responses.

### Self-Referential Memory Task

Before the VPT, participants completed the Reading the Mind in the Eyes Test (RMET; Baron-Cohen, Wheelwright, Hill, et al., 2001a). The task requires inferring a person’s mental state solely from the eye region using a four-choice multiple option with a control task requiring the identification of age and sex (Young Male, Young Female, Older Male, Older Female). To manipulate the self or other encoding of the memory for the mental attribute, following each choice, the participants were asked how often they felt that way (self-encoded) or how often they thought Barack Obama felt that way (other-encoded). Before the RMET, participants were shown a 5-minute documentary about Barack Obama to ensure familiarization. To encourage engagement with the task, participants were told that their responses would be compared against data collected from people who had worked with Barack Obama. The RMET also was used as a measure of baseline affective ToM/ emotion recognition (Table 3).

Following the VPT, participants performed a recognition memory task for the mental attribution words from the RMET. The correct mental attribution words as well as 76 distractor words (38 incorrect choices from the RMET and 38 novel words not previously seen) were presented and participants answered whether they had seen the mental attribution in the RMET task completed earlier. Responses were: 1 = Definitely did; 2 = Probably did; 3 = Probably not; 4 = Definitely not. Scoring was from 2 for a correct confident response through to −2 for a confident response that was incorrect. Words were divided according to whether they had been encoded in relation to the “self” or to the “other” (Barack Obama) and mean confidence scores were calculated.

### Source Memory Task

If participants responded that they had seen the mental attribution in the eyes, they were asked a subsequent question: “Was it on a male or a female face?” Responses were: 1 = Definitely male; 2 = Probably male; 3 = Probably female; 4 = Definitely female. Scoring was identical to the mental attribution memory task. This was considered a source memory, as it was a measure of a contextual memory not directly encoded in relation to the self or other.

For a detailed description of all tasks and stimulation procedures, please see Martin et al. (2017a, b, 2018). A schematic of the visual perspective taking tasks is presented in Fig. 1.

### Adverse Effects and Blinding

Adverse effects were assessed at the end of each stimulation session. Mood was assessed before and after each stimulation session (Brunoni et al., 2011) using the Visual Analogue of Mood Scale (VAMS; Folstein & Luria, 1973). Participant blinding was assessed by asking the participant to guess the active session following the completion of the study.

### Statistical Analysis

All analyses were conducted using JASP version 0.8.6. We applied a Bayesian approach alongside a frequentist approach (Wagenmakers et al., 2018). The Bayesian approach disperses with the null hypothesis assumption, whereby the null is either rejected or retained. It instead provides evidence for either the null or an alternate model and is presented in a continuous scale reflecting the strength of evidence. A Bayes Factor (BF) quantifies the evidence for a particular model. For example, a BF of 10 indicates that the data is 10 times more likely under that model and should be considered strong evidence. Although not entirely synonymous, a *p* value close to 0.05 is likely to only provide weak or preliminary evidence for either model. In this sense, a Bayesian approach moves away from dichotomous acceptance or rejection of the null model and enables more informed decision making based on uncertainty. Strength of evidence should be considered in a continuous manner, however for ease of interpretation we consider a BF between 1-3 to be preliminary evidence for the alternate model, 3-10 as moderate, and greater than 10 as strong evidence. Evidence for the null model follows the inverse pattern with a BF between 1-0.3 considered preliminary evidence, 0.3-0.1 as moderate, and less than 0.1 as strong evidence (Wagenmakers et al., 2018). We employed default priors for all analyses in JASP as recommended (Wagenmakers et al., 2018). Effect sizes are presented in the form of delta (*δ*) in the figures and text. Partial eta-squared (*η*^2^_p_) are used for ANOVA effect sizes.

Response times for all VPT measures were the variable of interest as the tasks were designed to keep errors low. The main effect of interest was the congruency effect, calculated by subtracting congruent from incongruent RTs as in previous studies (2019a, b; Martin et al., 2018). This is an index of the influence of the alternate perspective and was calculated for both egocentric and allocentric perspective judgements. For the implicit VPT task, we were interested in the different congruency effect of the avatar and the traffic light. For the explicit task this was not of interest and there was no different effect on congruency effect during level one egocentric judgements or allocentric judgements. Likewise, no difference was identified for level two egocentric judgements or allocentric judgements (see Martin, Perceval, et al., 2019a for more details).

Repeated measures ANOVAs were calculated for all tasks. For the implicit VPT, congruency effects for avatar and traffic light were within-subject factors and stimulation site was a between-subject factor. For both explicit VPT tasks, congruency effects (collapsed across agent) for both egocentric and allocentric conditions were within-subject factors and stimulation site was a between-subject factor. For the self-reference effect and source memory tasks, self and other encoded words was a within-subject factor and stimulation site was a between-subject factor. Stimulation (active and sham) was a within-subject factor across all tasks with order of stimulation counterbalanced across all tasks and both stimulation sites. All assumptions of ANOVA were satisfied. We present Bayesian ANOVA analyses alongside frequentist models. In all RM-ANOVA analyses, we initially report the model that best fits the data according to BF_10_ with the null model as comparison. To assess the contribution of each main effect or interaction, the relevant model is compared with or without the specific effect of interest, as per JASP guidelines (https://jasp-stats.org/).

Individual trials greater than 3 SDs from the overall mean were removed from all VPT tasks. Participants who failed to get 50% correct for any VPT condition were removed as it was deemed that they had not understood or completed the task according to instructions. At the dmPFC stimulation site, this resulted in the removal of 2 from the VPT level one and 3 from the level two. At the rTPJ stimulation site, two were removed from the VPT level one and three from the VPT level two. One older adult did not complete the SRE episodic memory task. Performance on all tasks during both stimulation conditions at each stimulation site is presented in Table 2.

## Results

### Visual Perspective Taking

#### Level One Explicit

**Fig. 2:**
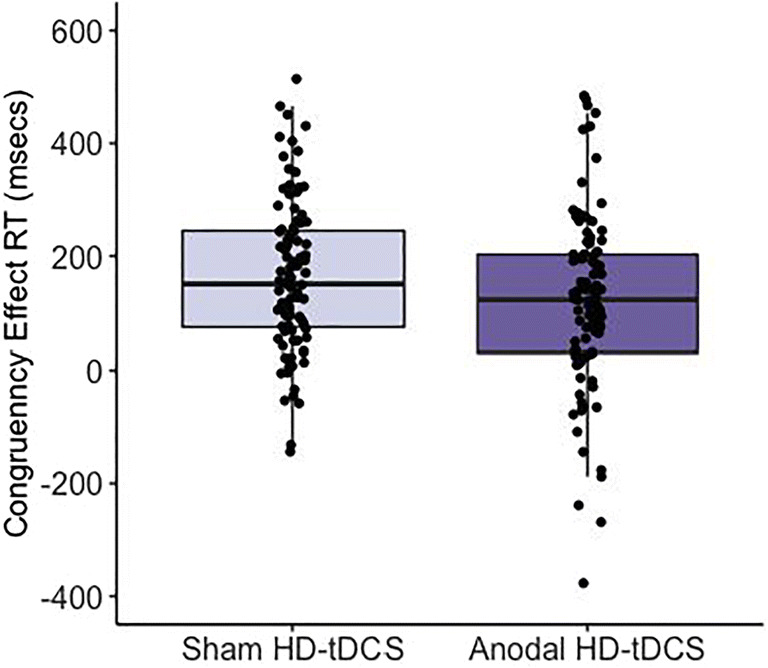
**Anodal stimulation to either the dmPFC or rTPJ reduced the congruency effect in both egocentric and allocentric conditions of the level one VPT (164.56 vs. 120.33 msec).** Data across both egocentric and allocentric conditions at both the dmPFC and rTPJ stimulation sites are presented. The boxplot represents the median and interquartile range (ICR). The whiskers extend to the most extreme datapoint ±1.5*IQR

A Bayesian Mixed-Factor ANOVA determined that the data are best represented by a model containing the main effects of PERSPECTIVE and STIMULATION. A BF_10_ = 6.29 indicates moderate support for this model compared with the null model (see Table S1 for full model statistics). Congruency effects were smaller during egocentric compared with allocentric perspective taking, F(1,46) = 5.23, *p* = 0.03, [BF_10_ = 3.23], *η*^2^_p_ = 0.10 (Table 1). An effect of Stimulation was supported, F(1,46) = 6.23, *p* = 0.02, [BF_10_ = 2.18], *η*^2^_p_ = 0.12 whereby anodal stimulation to either the dmPFC or rTPJ reduced the congruency effect (the influence of the alternate perspective) for both egocentric and allocentric perspective judgments (Fig. 2). The evidence supported the null model in regards to an interaction between Stimulation x Perspective, F(1,46) = 0.72, *p* = 0.40, [BF_10_ = 0.26], *η*^2^_p_ = 0.02 and for Stimulation x Stimulation Site, F(1,46) = 2.01, *p* = 0.16, [BF_10_ = 0.61], *η*^2^_p_ = 0.04. Likewise, the three-way interaction between Stimulation x Perspective x Stimulation Site favoured the null, F(1,46) = 0.99, *p* = 0.32, [BF_10_ = 0.46], *η*^2^_p_ = 0.02. Therefore, anodal stimulation reduced the congruency effect but this was not perspective specific, nor was it stimulation site-specific.

**Table 1:** Performance during sham and anodal HD-tDCS across all visual perspective taking tasks and the self-reference effect on episodic and source memory tasks

	dmPFC		rTPJ	
	Sham mean (SD)	Anodal mean (SD)	Sham mean (SD)	Anodal mean (SD)
Level one VPT				
*Ego CE*	107.61 (106.77)	91.21 (100.76)	189.51 (115.86)	86.92 (141.94)
*Allo CE*	161.99 (148.45)	140.19 (170.51)	199.13 (144.46)	163.00 (196.07)
Level two VPT				
*Ego CE*	306.22 (267.01)	275.31 (214.76)	300.88 (296.63)	325.48 (206.76)
*Allo CE*	188.79 (210.08)	145.03 (271.35)	254.42 (283.51)	171.36 (170.18)
Implicit VPT				
*Avatar CE*	20.87 (26.16)	10.97 (29.21)	2.45 (40.23)	20.81 (42.30)
*Light CE*	3.59 (24.92)	3.73 (36.46)	-7.39 (33.14)	-5.73 (35.18)
Episodic Memory				
*Self-encoded*	0.42 (0.68)	0.40 (0.61)	0.64 (0.55)	0.69 (0.60)
*Other-encoded*	0.37 (0.70)	0.27 (0.80)	0.52 (0.70)	0.56 (0.68)
Source Memory				
*Self-encoded*	0.40 (0.48)	0.35 (0.49)	0.27 (0.47)	0.32 (0.50)
*Other-encoded*	0.50 (0.50)	0.31 (0.60)	0.26 (0.46)	0.34 (0.37)

*dmPFC* dorsomedial prefrontal cortex, *rTPJ* right temporoparietal junction, *SD* standard deviation, *Ego* egocentric, *Allo* allocentric, *CE* congruency effect, *VPT* visual perspective taking

#### Level Two Explicit

A Bayesian Mixed-Factor ANOVA determined that the data are best represented by a model containing the main effect of PERSPECTIVE only. A BF_10_ = 31.58 indicates strong support for this model compared with the null model (see Table S2 for full model statistics). There was a significant main effect of Perspective, F(1,44) = 9.18, *p* = 0.004, [BF_10_ = 34.62], *η*^2^_p_ = 0.17, with greater congruency effects during egocentric compared with allocentric perspective taking (Table 1).The data supported the null model for a general stimulation effect, F(1,44) = 1.31, *p* = 0.26, [BF_10_ = 0.25], *η*^2^_p_ = 0.03. There was no interaction between Stimulation x Perspective, F(1,44) = 0.73, *p* = 0.40, [BF_10_ = 0.30], *η*^2^_p_ = 002 or for Stimulation x Stimulation Site, F(1,44) = 0.02, *p* = 0.89, [BF_10_ = 0.24], *η*^2^_p_ < 0.01. The three-way interaction between Stimulation x Perspective x Stimulation Site also supported the null model, F(1,44) = 0.45, *p* = 0.51, [BF_10_ = 0.36], *η*^2^_p_ = 0.01. In sum, anodal stimulation to either the dmPFC or the rTPJ had no effect on level two VPT.

#### Implicit VPT

A Bayesian Mixed-Factor ANOVA determined that the data are best represented by a model containing the main effect of AGENT only. BF_10_ = 26.88 indicates strong support for this model compared to the null model (see Table S3 for full model statistics). Strong support for an implicit VPT effect was identified by the main effect of Agent, F(1,50) = 11.90, *p* = 0.001, [BF_10_ = 27.35], *η*^2^_p_ = 0.19. There was support for the null model for all stimulation effects. Specifically, Stimulation, F(1,50) = 0.25, *p* = 0.62, [BF_10_ = 0.17], *η*^2^_p_ = 0.01, Stimulation x Agent, F(1,50) = 0.12, *p* = 0.73, [BF_10_ = 0.21], *η*^2^_p_ = 0.002, Stimulation x Stimulation Site, F(1,50) = 2.08, *p* = 0.16, [BF_10_ = 0.68], *η*^2^_p_ = 0.04, and the three-way interaction between Stimulation x Agent x Stimulation Site, F(1,50) = 1.98, *p* = 0.17, [BF_10_ = 0.70], *η*^2^_p_ = 0.04. In sum, participants were slower when the scene was incongruent with the avatar but not when a traffic light was in the scene. Anodal stimulation to the dmPFC or the rTPJ had no effect on implicit VPT.

### Self-Reference Effect on Episodic Memory

The null model was the best fit for the data (see Table S4 for full model statistics). No self-reference effect in episodic memory was identified, F(1,49) = 2.83, *p* = 0.10, [BF_10_ = 0.43], *η*^2^_p_ = 0.06. Stimulation had no effect on the SRE in episodic memory, F(1,49) = 0.12, *p* = 0.74, [BF_10_ = 0.22], *η*^2^_p_ = 0.002 and this was not site-specific, Stimulation x Stimulation Site x Agent, F(1,49) = 0.12, *p* = 0.73, [BF_10_ = 0.33], *η*^2^_p_ = 0.002. In sum, older adults did not remember self-encoded words better than other-encoded words and stimulation to either the dmPFC or rTPJ had no effect.

### Self-Reference Effect on Source Memory

The null model was the best fit for the data (see Table S5 for full model statistics). No self-reference effect for source memory was identified, F(1,49) = 0.09, *p* = 0.77, [BF_10_ = 0.16], *η*^2^_p_ = 0.002. Stimulation had no effect on SRE for source memory, F(1,49) = 0.18, *p* = 0.67, [BF_10_ = 0.22], *η*^2^_p_ = 0.004, and this was not site-specific, Stimulation x Stimulation Site x Agent, F(1,49) = 0.50, *p* = 0.48, [BF_10_ = 0.38], *η*^2^_p_ = 0.01. In sum, older adults did not remember source items in memory better for self-encoded items and stimulation to either the dmPFC or the rTPJ had no effect.

### Adverse Effects, Mood Change, and Blinding

Adverse effects and mood change are presented in Table 2. No change in positive mood change between stimulation sessions was identified, F(1,50) = 1.54, *p* = 0.22, [BF_10_ = 0.43], *η*^2^_p_ = 0.03, and there was no interaction with Stimulation Site, F(1,50) = 1.47, *p* = 0.23, [BF_10_ = 0.54], *η*^2^_p_ = 0.03. However, a slight increase in negative mood change was evident, F(1,50) = 4.10, *p* = 0.049, [BF_10_ = 1.19], *η*^2^_p_ = 0.08, and this interacted with Stimulation Site, F(1,50) = 4.23, *p* = 0.045, [BF_10_ = 1.78], *η*^2^_p_ = 0.08. Simple effects analysis identified an increase in negative mood after anodal dmPFC stimulation, t(25) = −2.04, *p* = 0.05, [BF_10_ = 1.23], *δ* = −0.48 and no change after rTPJ stimulation, t(25) = 0.25, *p* = 0.80, BF_10_ = 0.21, *δ* = 0.05. There was no difference in total adverse effects between sham and anodal stimulation sessions, F(1,50) < 0.001, *p* = 1.00, [BF_10_ = 0.20], *η*^2^_p_ < 0.001, and no interaction between Stimulation Type and Stimulation Site, F(1,50) = 0.27, *p* = 0.61, [BF_10_ = 0.33], *η*^2^_p_ = 0.01.

**Table 2:** Mood change and adverse effects for both stimulation sites

	dmPFC		rTPJ	
	Sham mean (sd)	Anodal mean (sd)	Sham Mean (sd)	Anodal mean (sd)
VAMS positive	0.84 (16.04)	−5.97 (25.00)	−1.01 (2.05)	−1.09 (2.37)
VAMS negative	0.94 (5.00)	4.79 (9.96)	0.11 (0.66)	0.07 (1.08)
Adverse effects	1.08 (1.44)	1.15 (1.85)	0.77 (0.95)	0.69 (1.23)

Only 23 of 52 guessed the stimulation order correctly, and this was comparable between stimulation sites (dmPFC/rTPJ: 12/11), χ^2^ = 0.08, *p* = 0.78.

Therefore, despite a small increase in negative mood after dmPFC stimulation, the stimulation effects are extremely unlikely to be due to mood change as stimulation at both sites improved VPT. Moreover, participants were unaware of the stimulation order demonstrating effective blinding.

### Baseline Cognition

All baseline cognitive performance is presented In Table 3. Cognitive differences between the older adults in the dmPFC and rTPJ groups were identified for the International Shopping List and one-back working memory. The rTPJ group outperformed the dmPFC group on the International Shopping List and vice-versa for the one-back working memory test. With the comparable performance on all other cognitive tests, it is extremely unlikely that any stimulation effects are influenced by baseline cognitive differences between the groups. Both groups were comparable on autistic traits, depression, and anxiety symptoms.

**Table 3:** Baseline cognitive performance for the dmPFC and rTPJ stimulation groups

	dmPFC Mean (sd)	rTPJ Mean (sd)	BF_10_
International shopping list	26.31 (4.38)	28.89 (3.47)	2.56
Identification task	2.76 (0.06)	2.78 (0.07)	0.51
One-back	2.90 (0.09)	2.94 (0.08)	1.54
Two-back	3.01 (0.10)	3.06 (0.10)	0.83
Set-switching errors	14.39 (6.32)	14.58 (5.52)	0.28
CPAL errors	79.81 (49.94)	78.42 (49.27)	0.28
Socio-emotional cognition	1.12 (0.11)	1.11 (0.12)	0.28
ISL – delayed	9.31 (2.00)	10.04 (1.82)	0.61
Phonemic fluency	16.5 (5.41)	16.65 (5.07)	0.28
Semantic fluency	20.96 (7.26)	22.77 (4.47)	0.45
Stroop effect	29.85 (10.50)	31.29 (13.73)	0.30
Reading the mind in the eyes	26.92 (3.67)	26.89 (3.80)	0.28
HADS depression	1.89 (2.60)	2.92 (3.22)	0.54
HADS anxiety	3.89 (2.22)	4.19 (2.84)	0.30
ASQ	15.62 (6.59)	13.85 (5.56)	0.44

*CPAL* Continuous Paired Associates Learning, *ISL* International Shopping List, *HADS* Hospital Anxiety and Depression Scale, *ASQ* Autism Spectrum Quotient

## Discussion

This is the first study that investigated regional and task-specific effects of HD-tDCS on self-other processing in older adults. Anodal stimulation to either the dmPFC and rTPJ improved self-other distinction as indexed by the reduced influence of the alternate perspective during line-of-sight (level one) visual perspective taking. Stimulation had no effect on embodied (level two) perspective taking nor the self-reference effect in episodic memory. These results differ to those identified in healthy young adults in previous replicated studies using identical tasks (Martin et al., 2018; Martin, Dzafic, et al., 2017a; Martin, Su, & Meinzer, 2019b), suggesting that age-associated brain reorganization may substantially affect stimulation effects.

Previous studies investigating tDCS effects in older adults have yielded mixed results. In some instances, stimulation of the same brain region resulted in similar cognitive effects in both young and older adults. However, some studies also showed positive stimulation effects in one age group only. Moreover, detrimental effects have been identified in older adults when using montages that yielded positive effects in young adults (see Perceval et al., 2016 for further discussion). In the present study, the first points of difference were the different effects on cognition and the lack of dissociable effects of dmPFC and rTPJ stimulation. In young adults, dmPFC stimulation increased the influence of the allocentric perspective during level one VPT, whereas in older adults we found that dmPFC stimulation reduced the influence of the alternate perspective regardless of the perspective taken (egocentric or allocentric). In young adults, rTPJ stimulation had a specific and dissociable effect of reducing the egocentric interference during a level two perspective taking task. This effect was not identified in older adults but rather, rTPJ stimulation had a similar effect as dmPFC stimulation in reducing the influence of the alternate perspective regardless of the perspective taken and for the level one perspective taking task only.

The results of the present study provide evidence that the role of specific social brain regions may change across the healthy lifespan. The effects of dmPFC and rTPJ stimulation in older adults were specific to perspective selection, rather than the dissociable effects observed in young adults. This suggests that these core social brain regions (Schurz et al., 2014) may have dissociable and specific causal roles in early adulthood but over the course of the natural lifespan, become less specific to certain cognitive components relevant to social functioning. Perspective selection is thought to rely on domain-general executive processes. For example, perspective-taking in a communication task was found to correlate with inhibition and switching ability across the healthy lifespan (Long et al., 2018). More specific to visual perspective-taking, Qureshi et al. (2010) found that performing a concurrent executive task impaired perspective selection during a level one VPT. Frontoparietal networks, including the dorsolateral prefrontal cortex and posterior parietal regions, have been associated with perspective selection (Ramsey et al., 2013). The present study supports this evidence and provides the first evidence of improving perspective selection in older adults using HD-tDCS to frontal and parietal regions. The previous imaging study by Ramsey et al. (2013) identified a frontoparietal network, including dorsolateral prefrontal cortex and temporoparietal junctions, involved in perspective selection in young adults. The fact that dmPFC stimulation improved perspective selection in older adults in the present study suggests that older adults may also recruit this brain region to inhibit the alternate perspective. One tentative explanation is age-related dedifferentiation of brain-behaviour associations (Baltes & Lindenberger, 1997; Li & Lindenberger, 1999). Further studies investigating perspective taking in older adults using functional neuroimaging will advance our understanding of age-related differences in stimulation response.

In addition to the different effects of stimulation on level one perspective taking, there was an absence of effect on level two perspective taking. The two tasks are thought to differ in respect to the requirement to perform an embodied rotation into the allocentric perspective. In young adults, rTPJ stimulation reduced the interference from the egocentric perspective during the embodied level two VPT task. The lack of stimulation effects in older adults may reflect different strategies employed by young and older adults. For example, it has been suggested that older adults may adopt less embodied strategies (Costello & Bloesch, 2017). Moreover, aging is associated with a general decline in mental imagery (Craik & Dirkx, 1992), often indexed by reduced accuracy on mental rotation tasks. However, more specific to embodied processes, older adults showed a greater deficit when the mental rotation task involved whole-body shapes (Devlin & Wilson, 2010) and egocentric rotations of their own body into that of another (Jansen & Kaltner, 2014; Kaltner & Jansen, 2016). It is therefore possible that bodily inputs have less effect on cognition in older adults and if the rTPJ is the seat of embodied processes regarding the body schema (Arzy et al., 2006), then stimulation of this region would not affect cognition in a comparable manner to that observed in younger adults. How embodied factors influence cognitive change across the lifespan is an ongoing research endeavour (Costello & Bloesch, 2017) and further research investigating age-related changes in brain-behaviour associations relevant to embodied cognition is warranted.

Finally, the absence of a self-reference effect in episodic memory in older adults corroborates previous research (Gutchess et al., 2015; Gutchess, Kensinger, Yoon, & Schacter, 2007b). In the present study, we provide novel evidence that, unlike in young adults, dmPFC stimulation had no effect on the self-reference effect. Neuroimaging evidence suggests a demarcation between vmPFC and dmPFC for self-other processing in younger adults (Denny et al., 2012; Northoff et al., 2006). Although this analysis has not been conducted in older adults, studies have shown functional brain differences in response to self-referential information (Gutchess, Kensinger, & Schacter, 2007a; Kalenzaga et al., 2014). Therefore, the lack of a SRE in episodic memory may reflect neural reorganisation and reduced demarcation between vmPFC and dmPFC. This also may explain the lack of stimulation effect and provides further evidence that baseline performance is an important consideration for subsequent stimulation effects (Berryhill & Jones, 2012; Learmonth et al., 2015; Meinzer et al., 2013). For example, in our previous study (Martin, Su, & Meinzer, 2019b), we identified baseline differences on visual perspective taking in a cohort of South-East Asian participants. Stimulation effects were comparable with the Caucasian cohort across all tasks, except the VPT task with baseline differences. The results from the present study provide further evidence that baseline performance is an important consideration for predicting subsequent stimulation effects.

The ability to distinguish between self and other has been measured in a number of ways (Hamilton et al., 2014). The results of the current study may not generalize to other tasks. Future research is required to ascertain whether baseline differences in executive and social abilities mediate the stimulation effects in older adults and helps to explains the different effects as those observed in younger adults (Martin et al., 2018). Likewise, direct comparison of HD-tDCS effects between different age groups and the mediating role of baseline cognition will be achievable with larger samples. Alternate stimulation sites may also provide insights into age-related differences in causal brain-behaviour associations for perspective taking in older adults. For example, the inferior frontal gyri have been causally associated with processes relevant to self-other processing, including the social categorization of space (Fini et al., 2017), interpersonal motor resonance (Enticott et al., 2012), and empathy (Peled-Avron et al., 2019). Despite nonsignificant effects of cathodal stimulation to the dmPFC on self-other processing in young adults (Martin, Dzafic, et al., 2017a), research into effects in older adults is lacking. Multisession tDCS has been effective in improving cognition in older adults over longer timescales (Antonenko et al., 2018; Jones et al., 2015; Park et al., 2014; Perceval et al., 2020; Stephens & Berryhill, 2016) and should motivate future research in the social domain. We also included older adults with a range of ages from 55-79 years. This represents a wide range of cognitive aging effects, and stimulation effects may differ in a continuous manner across healthy aging.

In sum, anodal stimulation to the dmPFC and rTPJ improved perspective selection during level one VPT in older adults by reducing the influence of the nontask relevant perspective during both egocentric and allocentric perspective judgements. The results provide novel causal evidence for the role of key social brain regions in self-other processing in older adults and highlights differential stimulation effects across the human lifespan.

## Supplementary Information

ESM 1 (DOCX 42 kb)
