# Supplementary material for: Stimulation of the Social Brain Improves Perspective Selection in Older Adults: A HD-tDCS Study
Source: Cogn Affect Behav Neurosci. 2021 Jul 21;21(6):1233–45. doi: 10.3758/s13415-021-00929-2 (PMC8563543; doi:10.3758/s13415-021-00929-2)
Supplement: Supplementary file 1 — (DOCX 42 kb) [file 13415_2021_929_MOESM1_ESM.docx]

Table S1. Bayesian model comparison for Level 1 Visual Perspective Taking

| **Model Comparison** | | | | | | | | | | |  |
| --- | --- | --- | --- | --- | --- | --- | --- | --- | --- | --- | --- |
| **Models** | | **P(M)** | | **P(M\|data)** | | **BF _M_** | | **BF _10_** | | **error %** |  |
| Null model (incl. subject) |  | 0.053 |  | 0.041 |  | 0.773 |  | 1.000 |  |  |  |
| stim |  | 0.053 |  | 0.080 |  | 1.567 |  | 1.946 |  | 0.917 |  |
| perspective |  | 0.053 |  | 0.118 |  | 2.412 |  | 2.871 |  | 1.208 |  |
| stim + perspective |  | 0.053 |  | 0.259 |  | 6.286 |  | 6.290 |  | 2.282 |  |
| stim + perspective + stim  ✻  perspective |  | 0.053 |  | 0.073 |  | 1.423 |  | 1.780 |  | 3.145 |  |
| StimSite |  | 0.053 |  | 0.018 |  | 0.324 |  | 0.430 |  | 1.475 |  |
| stim + StimSite |  | 0.053 |  | 0.037 |  | 0.689 |  | 0.896 |  | 3.366 |  |
| perspective + StimSite |  | 0.053 |  | 0.050 |  | 0.945 |  | 1.212 |  | 1.239 |  |
| stim + perspective + StimSite |  | 0.053 |  | 0.115 |  | 2.341 |  | 2.797 |  | 3.588 |  |
| stim + perspective + stim  ✻  perspective + StimSite |  | 0.053 |  | 0.032 |  | 0.592 |  | 0.774 |  | 2.664 |  |
| stim + StimSite + stim  ✻  StimSite |  | 0.053 |  | 0.016 |  | 0.301 |  | 0.400 |  | 2.217 |  |
| stim + perspective + StimSite + stim  ✻  StimSite |  | 0.053 |  | 0.080 |  | 1.560 |  | 1.938 |  | 24.422 |  |
| stim + perspective + stim  ✻  perspective + StimSite + stim  ✻  StimSite |  | 0.053 |  | 0.015 |  | 0.279 |  | 0.371 |  | 2.413 |  |
| perspective + StimSite + perspective  ✻  StimSite |  | 0.053 |  | 0.011 |  | 0.200 |  | 0.268 |  | 2.062 |  |
| stim + perspective + StimSite + perspective  ✻  StimSite |  | 0.053 |  | 0.027 |  | 0.491 |  | 0.645 |  | 8.168 |  |
| stim + perspective + stim  ✻  perspective + StimSite + perspective  ✻  StimSite |  | 0.053 |  | 0.006 |  | 0.116 |  | 0.156 |  | 2.267 |  |
| stim + perspective + StimSite + stim  ✻  StimSite + perspective  ✻  StimSite |  | 0.053 |  | 0.017 |  | 0.311 |  | 0.413 |  | 27.036 |  |
| stim + perspective + stim  ✻  perspective + StimSite + stim  ✻  StimSite + perspective  ✻  StimSite |  | 0.053 |  | 0.003 |  | 0.058 |  | 0.079 |  | 3.119 |  |
| stim + perspective + stim  ✻  perspective + StimSite + stim  ✻  StimSite + perspective  ✻  StimSite + stim  ✻  perspective  ✻  StimSite |  | 0.053 |  | 0.001 |  | 0.027 |  | 0.036 |  | 5.102 |  |
|  | | | | | | | | | | |  |
| *Note.*  All models include subject. | | | | | | | | | | |  |

| **Analysis of Effects** | | | | | | | |
| --- | --- | --- | --- | --- | --- | --- | --- |
| **Effects** | | **P(incl)** | | **P(incl\|data)** | | **BF _Inclusion_** | |
| stim |  | 0.263 |  | 0.517 |  | 2.175 |  |
| perspective |  | 0.263 |  | 0.622 |  | 3.233 |  |
| StimSite |  | 0.263 |  | 0.251 |  | 0.440 |  |
| stim  ✻  perspective |  | 0.263 |  | 0.130 |  | 0.261 |  |
| stim  ✻  StimSite |  | 0.263 |  | 0.132 |  | 0.608 |  |
| perspective  ✻  StimSite |  | 0.263 |  | 0.064 |  | 0.220 |  |
| stim  ✻  perspective  ✻  StimSite |  | 0.053 |  | 0.001 |  | 0.457 |  |
|  | | | | | | | |
| *Note.*  Compares models that contain the effect to equivalent models stripped of the effect. Higher-order interactions are excluded. Analysis suggested by Sebastiaan Mathôt. | | | | | | | |

Table S2. Bayesian model comparison for level 2 Visual Perspective Taking

| **Model Comparison** | | | | | | | | | | | |
| --- | --- | --- | --- | --- | --- | --- | --- | --- | --- | --- | --- |
| **Models** | | **P(M)** | | **P(M\|data)** | | **BF _M_** | | **BF _10_** | | **error %** | |
| Null model (incl. subject) |  | 0.053 |  | 0.017 |  | 0.309 |  | 1.000 |  |  |  |
| Stim |  | 0.053 |  | 0.004 |  | 0.075 |  | 0.246 |  | 0.876 |  |
| Persp |  | 0.053 |  | 0.533 |  | 20.523 |  | 31.576 |  | 0.994 |  |
| Stim + Persp |  | 0.053 |  | 0.143 |  | 2.997 |  | 8.461 |  | 4.225 |  |
| Stim + Persp + Stim  ✻  Persp |  | 0.053 |  | 0.045 |  | 0.843 |  | 2.653 |  | 3.185 |  |
| StimSite |  | 0.053 |  | 0.004 |  | 0.080 |  | 0.262 |  | 0.855 |  |
| Stim + StimSite |  | 0.053 |  | 0.001 |  | 0.020 |  | 0.065 |  | 1.631 |  |
| Persp + StimSite |  | 0.053 |  | 0.144 |  | 3.040 |  | 8.564 |  | 1.415 |  |
| Stim + Persp + StimSite |  | 0.053 |  | 0.037 |  | 0.696 |  | 2.206 |  | 1.877 |  |
| Stim + Persp + Stim  ✻  Persp + StimSite |  | 0.053 |  | 0.012 |  | 0.214 |  | 0.695 |  | 4.782 |  |
| Stim + StimSite + Stim  ✻  StimSite |  | 0.053 |  | 2.363e -4 |  | 0.004 |  | 0.014 |  | 2.743 |  |
| Stim + Persp + StimSite + Stim  ✻  StimSite |  | 0.053 |  | 0.008 |  | 0.153 |  | 0.499 |  | 3.792 |  |
| Stim + Persp + Stim  ✻  Persp + StimSite + Stim  ✻  StimSite |  | 0.053 |  | 0.002 |  | 0.045 |  | 0.147 |  | 3.933 |  |
| Persp + StimSite + Persp  ✻  StimSite |  | 0.053 |  | 0.035 |  | 0.647 |  | 2.057 |  | 5.476 |  |
| Stim + Persp + StimSite + Persp  ✻  StimSite |  | 0.053 |  | 0.009 |  | 0.156 |  | 0.509 |  | 3.262 |  |
| Stim + Persp + Stim  ✻  Persp + StimSite + Persp  ✻  StimSite |  | 0.053 |  | 0.003 |  | 0.050 |  | 0.165 |  | 5.878 |  |
| Stim + Persp + StimSite + Stim  ✻  StimSite + Persp  ✻  StimSite |  | 0.053 |  | 0.002 |  | 0.033 |  | 0.108 |  | 2.872 |  |
| Stim + Persp + Stim  ✻  Persp + StimSite + Stim  ✻  StimSite + Persp  ✻  StimSite |  | 0.053 |  | 5.303e -4 |  | 0.010 |  | 0.031 |  | 3.143 |  |
| Stim + Persp + Stim  ✻  Persp + StimSite + Stim  ✻  StimSite + Persp  ✻  StimSite + Stim  ✻  Persp  ✻  StimSite |  | 0.053 |  | 2.142e -4 |  | 0.004 |  | 0.013 |  | 4.638 |  |
|  | | | | | | | | | | | |
| *Note.*  All models include subject. | | | | | | | | | | | |

| **Analysis of Effects** | | | | | | | |
| --- | --- | --- | --- | --- | --- | --- | --- |
| **Effects** | | **P(incl)** | | **P(incl\|data)** | | **BF _Inclusion_** | |
| Stim |  | 0.263 |  | 0.194 |  | 0.264 |  |
| Persp |  | 0.263 |  | 0.866 |  | 32.316 |  |
| StimSite |  | 0.263 |  | 0.199 |  | 0.268 |  |
| Stim  ✻  Persp |  | 0.263 |  | 0.062 |  | 0.313 |  |
| Stim  ✻  StimSite |  | 0.263 |  | 0.013 |  | 0.220 |  |
| Persp  ✻  StimSite |  | 0.263 |  | 0.048 |  | 0.237 |  |
| Stim  ✻  Persp  ✻  StimSite |  | 0.053 |  | 2.142e -4 |  | 0.404 |  |
|  | | | | | | | |
| *Note.*  Compares models that contain the effect to equivalent models stripped of the effect. Higher-order interactions are excluded. Analysis suggested by Sebastiaan Mathôt. | | | | | | | |

Table S3. Bayesian model comparison for Implicit Perspective Taking

| **Model Comparison** | | | | | | | | | | | |
| --- | --- | --- | --- | --- | --- | --- | --- | --- | --- | --- | --- |
| **Models** | | **P(M)** | | **P(M\|data)** | | **BF _M_** | | **BF _10_** | | **error %** | |
| Null model (incl. subject) |  | 0.053 |  | 0.019 |  | 0.352 |  | 1.000 |  |  |  |
| stim |  | 0.053 |  | 0.003 |  | 0.060 |  | 0.173 |  | 1.556 |  |
| agent |  | 0.053 |  | 0.516 |  | 19.155 |  | 26.884 |  | 1.208 |  |
| stim + agent |  | 0.053 |  | 0.091 |  | 1.798 |  | 4.736 |  | 1.898 |  |
| stim + agent + stim  ✻  agent |  | 0.053 |  | 0.019 |  | 0.340 |  | 0.967 |  | 1.587 |  |
| StimSite |  | 0.053 |  | 0.007 |  | 0.129 |  | 0.372 |  | 0.928 |  |
| stim + StimSite |  | 0.053 |  | 0.001 |  | 0.022 |  | 0.064 |  | 1.511 |  |
| agent + StimSite |  | 0.053 |  | 0.202 |  | 4.546 |  | 10.515 |  | 2.448 |  |
| stim + agent + StimSite |  | 0.053 |  | 0.034 |  | 0.635 |  | 1.777 |  | 1.991 |  |
| stim + agent + stim  ✻  agent + StimSite |  | 0.053 |  | 0.008 |  | 0.137 |  | 0.395 |  | 2.628 |  |
| stim + StimSite + stim  ✻  StimSite |  | 0.053 |  | 7.975e -4 |  | 0.014 |  | 0.042 |  | 2.046 |  |
| stim + agent + StimSite + stim  ✻  StimSite |  | 0.053 |  | 0.023 |  | 0.430 |  | 1.216 |  | 2.140 |  |
| stim + agent + stim  ✻  agent + StimSite + stim  ✻  StimSite |  | 0.053 |  | 0.005 |  | 0.089 |  | 0.258 |  | 2.730 |  |
| agent + StimSite + agent  ✻  StimSite |  | 0.053 |  | 0.053 |  | 1.017 |  | 2.790 |  | 3.358 |  |
| stim + agent + StimSite + agent  ✻  StimSite |  | 0.053 |  | 0.009 |  | 0.155 |  | 0.444 |  | 3.725 |  |
| stim + agent + stim  ✻  agent + StimSite + agent  ✻  StimSite |  | 0.053 |  | 0.002 |  | 0.034 |  | 0.097 |  | 5.898 |  |
| stim + agent + StimSite + stim  ✻  StimSite + agent  ✻  StimSite |  | 0.053 |  | 0.006 |  | 0.104 |  | 0.298 |  | 2.879 |  |
| stim + agent + stim  ✻  agent + StimSite + stim  ✻  StimSite + agent  ✻  StimSite |  | 0.053 |  | 0.001 |  | 0.024 |  | 0.070 |  | 5.284 |  |
| stim + agent + stim  ✻  agent + StimSite + stim  ✻  StimSite + agent  ✻  StimSite + stim  ✻  agent  ✻  StimSite |  | 0.053 |  | 9.428e -4 |  | 0.017 |  | 0.049 |  | 7.102 |  |
|  | | | | | | | | | | | |
| *Note.*  All models include subject. | | | | | | | | | | | |

| **Analysis of Effects** | | | | | | | |
| --- | --- | --- | --- | --- | --- | --- | --- |
| **Effects** | | **P(incl)** | | **P(incl\|data)** | | **BF _Inclusion_** | |
| stim |  | 0.263 |  | 0.138 |  | 0.173 |  |
| agent |  | 0.263 |  | 0.865 |  | 27.352 |  |
| StimSite |  | 0.263 |  | 0.252 |  | 0.389 |  |
| stim  ✻  agent |  | 0.263 |  | 0.034 |  | 0.211 |  |
| stim  ✻  StimSite |  | 0.263 |  | 0.036 |  | 0.678 |  |
| agent  ✻  StimSite |  | 0.263 |  | 0.071 |  | 0.261 |  |
| stim  ✻  agent  ✻  StimSite |  | 0.053 |  | 9.428e -4 |  | 0.700 |  |
|  | | | | | | | |
| *Note.*  Compares models that contain the effect to equivalent models stripped of the effect. Higher-order interactions are excluded. Analysis suggested by Sebastiaan Mathôt. | | | | | | | |

Table S4. Bayesian model comparison for Self-reference effect in episodic memory

| **Model Comparison** | | | | | | | | | | | |
| --- | --- | --- | --- | --- | --- | --- | --- | --- | --- | --- | --- |
| **Models** | | **P(M)** | | **P(M\|data)** | | **BF _M_** | | **BF _10_** | | **error %** | |
| Null model (incl. subject) |  | 0.053 |  | 0.296 |  | 7.560 |  | 1.000 |  |  |  |
| Stim |  | 0.053 |  | 0.044 |  | 0.835 |  | 0.150 |  | 0.980 |  |
| Agent |  | 0.053 |  | 0.150 |  | 3.188 |  | 0.509 |  | 1.359 |  |
| Stim + Agent |  | 0.053 |  | 0.022 |  | 0.405 |  | 0.074 |  | 1.441 |  |
| Stim + Agent + Stim  ✻  Agent |  | 0.053 |  | 0.005 |  | 0.087 |  | 0.016 |  | 3.613 |  |
| StimSite |  | 0.053 |  | 0.251 |  | 6.036 |  | 0.849 |  | 1.762 |  |
| Stim + StimSite |  | 0.053 |  | 0.038 |  | 0.707 |  | 0.128 |  | 1.749 |  |
| Agent + StimSite |  | 0.053 |  | 0.122 |  | 2.500 |  | 0.412 |  | 1.157 |  |
| Stim + Agent + StimSite |  | 0.053 |  | 0.019 |  | 0.352 |  | 0.065 |  | 5.401 |  |
| Stim + Agent + Stim  ✻  Agent + StimSite |  | 0.053 |  | 0.004 |  | 0.073 |  | 0.014 |  | 2.889 |  |
| Stim + StimSite + Stim  ✻  StimSite |  | 0.053 |  | 0.010 |  | 0.181 |  | 0.034 |  | 1.627 |  |
| Stim + Agent + StimSite + Stim  ✻  StimSite |  | 0.053 |  | 0.006 |  | 0.101 |  | 0.019 |  | 6.674 |  |
| Stim + Agent + Stim  ✻  Agent + StimSite + Stim  ✻  StimSite |  | 0.053 |  | 0.001 |  | 0.021 |  | 0.004 |  | 4.366 |  |
| Agent + StimSite + Agent  ✻  StimSite |  | 0.053 |  | 0.026 |  | 0.472 |  | 0.086 |  | 1.842 |  |
| Stim + Agent + StimSite + Agent  ✻  StimSite |  | 0.053 |  | 0.004 |  | 0.072 |  | 0.013 |  | 3.614 |  |
| Stim + Agent + Stim  ✻  Agent + StimSite + Agent  ✻  StimSite |  | 0.053 |  | 8.923e -4 |  | 0.016 |  | 0.003 |  | 6.989 |  |
| Stim + Agent + StimSite + Stim  ✻  StimSite + Agent  ✻  StimSite |  | 0.053 |  | 0.001 |  | 0.020 |  | 0.004 |  | 3.291 |  |
| Stim + Agent + Stim  ✻  Agent + StimSite + Stim  ✻  StimSite + Agent  ✻  StimSite |  | 0.053 |  | 2.296e -4 |  | 0.004 |  | 7.762e -4 |  | 3.905 |  |
| Stim + Agent + Stim  ✻  Agent + StimSite + Stim  ✻  StimSite + Agent  ✻  StimSite + Stim  ✻  Agent  ✻  StimSite |  | 0.053 |  | 7.656e -5 |  | 0.001 |  | 2.589e -4 |  | 7.700 |  |
|  | | | | | | | | | | | |
| *Note.*  All models include subject. | | | | | | | | | | | |

| **Analysis of Effects** | | | | | | | |
| --- | --- | --- | --- | --- | --- | --- | --- |
| **Effects** | | **P(incl)** | | **P(incl\|data)** | | **BF _Inclusion_** | |
| Stim |  | 0.263 |  | 0.127 |  | 0.151 |  |
| Agent |  | 0.263 |  | 0.319 |  | 0.499 |  |
| StimSite |  | 0.263 |  | 0.434 |  | 0.839 |  |
| Stim  ✻  Agent |  | 0.263 |  | 0.011 |  | 0.215 |  |
| Stim  ✻  StimSite |  | 0.263 |  | 0.018 |  | 0.273 |  |
| Agent  ✻  StimSite |  | 0.263 |  | 0.032 |  | 0.209 |  |
| Stim  ✻  Agent  ✻  StimSite |  | 0.053 |  | 7.656e -5 |  | 0.333 |  |
|  | | | | | | | |
| *Note.*  Compares models that contain the effect to equivalent models stripped of the effect. Higher-order interactions are excluded. Analysis suggested by Sebastiaan Mathôt. | | | | | | | |

Table S5. Bayesian model comparison for Self-reference effect in source memory

| **Model Comparison** | | | | | | | | | | | |
| --- | --- | --- | --- | --- | --- | --- | --- | --- | --- | --- | --- |
| **Models** | | **P(M)** | | **P(M\|data)** | | **BF _M_** | | **BF _10_** | | **error %** | |
| Null model (incl. subject) |  | 0.053 |  | 0.520 |  | 19.499 |  | 1.000 |  |  |  |
| Stim |  | 0.053 |  | 0.088 |  | 1.741 |  | 0.170 |  | 0.915 |  |
| Agent |  | 0.053 |  | 0.084 |  | 1.653 |  | 0.162 |  | 1.531 |  |
| Stim + Agent |  | 0.053 |  | 0.014 |  | 0.257 |  | 0.027 |  | 2.215 |  |
| Stim + Agent + Stim  ✻  Agent |  | 0.053 |  | 0.003 |  | 0.058 |  | 0.006 |  | 5.686 |  |
| StimSite |  | 0.053 |  | 0.189 |  | 4.188 |  | 0.363 |  | 1.674 |  |
| Stim + StimSite |  | 0.053 |  | 0.032 |  | 0.589 |  | 0.061 |  | 2.435 |  |
| Agent + StimSite |  | 0.053 |  | 0.029 |  | 0.537 |  | 0.056 |  | 1.775 |  |
| Stim + Agent + StimSite |  | 0.053 |  | 0.005 |  | 0.093 |  | 0.010 |  | 2.530 |  |
| Stim + Agent + Stim  ✻  Agent + StimSite |  | 0.053 |  | 0.001 |  | 0.021 |  | 0.002 |  | 2.703 |  |
| Stim + StimSite + Stim  ✻  StimSite |  | 0.053 |  | 0.022 |  | 0.401 |  | 0.042 |  | 5.969 |  |
| Stim + Agent + StimSite + Stim  ✻  StimSite |  | 0.053 |  | 0.003 |  | 0.058 |  | 0.006 |  | 2.496 |  |
| Stim + Agent + Stim  ✻  Agent + StimSite + Stim  ✻  StimSite |  | 0.053 |  | 8.338e -4 |  | 0.015 |  | 0.002 |  | 8.372 |  |
| Agent + StimSite + Agent  ✻  StimSite |  | 0.053 |  | 0.006 |  | 0.111 |  | 0.012 |  | 3.502 |  |
| Stim + Agent + StimSite + Agent  ✻  StimSite |  | 0.053 |  | 0.002 |  | 0.030 |  | 0.003 |  | 42.753 |  |
| Stim + Agent + Stim  ✻  Agent + StimSite + Agent  ✻  StimSite |  | 0.053 |  | 2.366e -4 |  | 0.004 |  | 4.550e -4 |  | 5.543 |  |
| Stim + Agent + StimSite + Stim  ✻  StimSite + Agent  ✻  StimSite |  | 0.053 |  | 6.795e -4 |  | 0.012 |  | 0.001 |  | 4.030 |  |
| Stim + Agent + Stim  ✻  Agent + StimSite + Stim  ✻  StimSite + Agent  ✻  StimSite |  | 0.053 |  | 1.442e -4 |  | 0.003 |  | 2.773e -4 |  | 3.167 |  |
| Stim + Agent + Stim  ✻  Agent + StimSite + Stim  ✻  StimSite + Agent  ✻  StimSite + Stim  ✻  Agent  ✻  StimSite |  | 0.053 |  | 5.509e -5 |  | 9.916e -4 |  | 1.059e -4 |  | 4.241 |  |
|  | | | | | | | | | | | |
| Note.  All models include subject. | | | | | | | | | | | |

| **Analysis of Effects** | | | | | | | |
| --- | --- | --- | --- | --- | --- | --- | --- |
| **Effects** | | **P(incl)** | | **P(incl\|data)** | | **BF _Inclusion_** | |
| Stim |  | 0.263 |  | 0.141 |  | 0.170 |  |
| Agent |  | 0.263 |  | 0.136 |  | 0.159 |  |
| StimSite |  | 0.263 |  | 0.256 |  | 0.360 |  |
| Stim  ✻  Agent |  | 0.263 |  | 0.006 |  | 0.224 |  |
| Stim  ✻  StimSite |  | 0.263 |  | 0.027 |  | 0.667 |  |
| Agent  ✻  StimSite |  | 0.263 |  | 0.009 |  | 0.226 |  |
| Stim  ✻  Agent  ✻  StimSite |  | 0.053 |  | 5.509e -5 |  | 0.382 |  |
|  | | | | | | | |
| Note.  Compares models that contain the effect to equivalent models stripped of the effect. Higher-order interactions are excluded. Analysis suggested by Sebastiaan Mathôt. | | | | | | | |
